# Comparative analysis of treatments for vertebrogenic chronic low back pain: Intradiscal steroid injections vs. basivertebral nerve radiofrequency ablation

**DOI:** 10.3892/mi.2025.275

**Published:** 2025-10-14

**Authors:** Evangelos Sakellariou, Panagiotis Karampinas, Athanasios Galanis, Evangelia Argyropoulou, Dimitris Tzortzis, Andreas Morakis, Nikolaos Parchas, Meletis Rozis, Vasileios Marougklianis, Angelos Kaspiris, Elias Vasiliadis, Spyros Pneumaticos

**Affiliations:** 1 Department of Orthopaedic Surgery, KAT General Hospital, National and Kapodistrian University of Athens, 14561 Athens, Greece; 2 Department of Orthopaedics and Traumatology, University General Hospital of Patras, 26504 Patras, Greece; 3 Interventional Radiology Department, KAT General Hospital, National and Kapodistrian University of Athens, 14561 Athens, Greece

**Keywords:** chronic low back pain, vertebrogenic, depo-methyl prednisolone injection, intradiscal steroid injection, basivertebral nerve radiofrequency ablation, pain management, Oswestry Disability Index, personalized treatment

## Abstract

Vertebrogenic chronic low back pain (CLBP) significantly impairs the quality of life of patients, necessitating effective treatment strategies. The present study compared two intervention strategies for this condition: Intradiscal steroid injection (IDSI) in one group with 12 patients, vs. intraosseous basivertebral nerve radiofrequency ablation (BVA) in another group of 10 patients. The present study included patients aged 18-70 years, irrespective of sex, whilst specific inclusion and exclusion criteria were applied. IDSI involves corticosteroid injections into the intervertebral disc, reducing inflammation and pain, whereas BVA targets the basivertebral nerve to disrupt pain signals. A retrospective analysis of the Oswestry Disability Index (ODI) scores of the patients before and after treatment revealed that both treatments effectively reduce disability. The IDSI revealed a higher percentage of complete symptom resolution (at 75%), whereas BVA appeared to provide more consistent and notable improvements across patients, with complete symptom resolution at 70%. Despite potential side-effects, such as transient pain, increased blood sugar levels, nerve damage or infection, both treatments provide substantial pain relief and benefits that outweigh typical side-effects, presumably underscoring the importance of personalized treatment plans. Nonetheless, taking into consideration the notable limitations of the present study, it is essential to emphasize the requirement for further larger-scale research.

## Introduction

Chronic low back pain (CLBP) is a pervasive condition that significantly impairs the quality of life of patients by limiting daily activities and overall well-being (1,2). Additionally, anterior column pain, the cause of CLBP, has been theorized to stem from the pain receptors of intervertebral discs. However, recent histological data suggest that the vertebral endplates may play a predominant role, as they manifest up to four times the number of nerve receptors compared with intervertebral discs (3-5).

Overall, vertebrogenic low back pain and discogenic low back pain are considered two entirely dissimilar entities. Discogenic pain arises from the degeneration of the intervertebral disc, particularly the annulus fibrosus, and is transmitted via the sinuvertebral nerve. Discogenic pain accounts for a substantial portion of cases of CLBP, with estimates ranging from 26 to 42% (2-5). It is caused by structural defects, such as annular tears, disc dehydration and the loss of disc height, leading to inflammation, nerve ingrowth and altered biomechanics. Clinically, it presents as axial low back pain worsened by sitting and flexion, with referred pain above the knees in a non-dermatomal pattern and no neurological deficits. Magnetic resonance imaging (MRI) may depict disc desiccation and high-intensity zones, whilst a discography can confirm diagnosis, although this is considered invasive (3-7). On the contrary, vertebrogenic pain is a newer concept linked to the damage and inflammation of the vertebral endplates, innervated by the basivertebral nerve. It is noteworthy that vertebrogenic pain is estimated to affect ~1 in 6 individuals with CLBP in the USA (3-6). It is associated with Modic type 1 or 2 alterations observed on an MRI and results from endplate microtrauma, allowing inflammatory disc material to infiltrate the bone marrow. Symptoms typically involve midline low back pain, which deteriorates in the morning or with activity, with possible referral to paraspinal or gluteal regions. Unlike discogenic pain, it is not aggravated by extension and may present without significant disc abnormalities on imaging (5-7). First-line management for CLBP, including vertebrogenic CLBP, involves nonpharmacologic therapy such as exercise, physical therapy, spinal manipulation and acupuncture, while non-steroidal anti-inflammatory drugs, muscle relaxants etc. are used as second-line therapy (7); however, in a significant portion of individuals, those treatments do not exhibit the desired outcomes (5-7). The main limitations of the conservative management of vertebrogenic CLBP are considered limited long-term effectiveness, the inability to address the source of pain, the risk of dependence on pain medication and the lack of improvement with certain therapies such as traction or transcutaneous electrical nerve stimulation (4-8).

In light of this evidence, interventions have been employed to manage CLBP by specifically targeting the intradiscal space and vertebral endplates, including intradiscal steroid injections (IDSIs) and intraosseous basivertebral nerve radiofrequency ablations (BVAs) (8,9). IDSI involves injecting corticosteroids in the center of intervertebral discs (10), whereas BVA cauterizes the basivertebral nerve within the vertebral body (11,12). There is clinical ambiguity as regards which of these two interventions is superior concerning the treatment of vertebrogenic CLBP. Targeting the basivertebral nerve may be more effective than targeting the disc itself for vertebrogenic pain, as the basivertebral nerve is the primary nerve responsible for transmitting pain signals from the vertebral endplates, which are frequently damaged in cases of vertebrogenic pain, and nerve density is higher in endplates (7-12). To the best of our knowledge, no study in the existing literature to date has performed a direct comparison of IDSI and BVA for the treatment of vertebrogenic CLBP. Thus, the present study aimed to fill this gap in the literature. The present study aimed to compare the efficacy of these two interventions in reducing disability and pain levels in patients with vertebrogenic CLBP, while utilizing the Oswestry Disability Index (ODI) (13), as it is specific for CLBP and easy to use. The present study aimed to provide clinicians with insight into the most effective treatment options for this challenging condition.

### Background information and description of IDSI and BVA techniques

During intradiscal steroid injections, a single physician performs each intradiscal injection via the posterolateral disc access approach. The patient lies in a prone position, and the targeted intervertebral disc is identified via fluoroscopy. The overlying skin is prepped surgically and anesthetized with lidocaine. A 22-gauge spinal needle with guidance from intermittent fluoroscopy is then advanced towards the center of the disc via a right or left posterolateral oblique window. Finally, anteroposterior and lateral visualization ensures the correct needle position before 2 cc of depo-methylprednisolone is inserted into the disc space. Depo-methylprednisolone is delivered directly into the intravertebral space, where it exerts potent anti-inflammatory and analgesic effects (14). This reduces inflammation, alleviates pain and improves functional outcomes in patients suffering from CLBP (15,16). IDSI has shown promising results, with a number of patients experiencing substantial reductions in disability levels post-injection (12,14). However, the outcomes can vary, with some patients demonstrating a complete resolution of symptoms and others exhibiting minimal improvement (12,14-19). Reported side-effects known from the literature include transient pain at the injection site, increased blood sugar levels, and, in rare cases, infection. These potential side-effects necessitate careful patient selection and monitoring (17-19).

On the other hand, during intraosseous basivertebral nerve radiofrequency ablation, the patient is in the prone position, the C-Arm is prepositioned, the skin is prepped surgically, and local anesthetization is applied. Multiple fluoroscopies are employed to ensure that the proper trajectory of the trocar in pedicle care starts at the superior lateral aspect until it passes through the posterior vertebral body wall. It is important to maintain a position superior to the inferior cortex and lateral to the medial cortex of the pedicle to prevent trocar entry into the spinal canal or near neural elements. Once the trocar is inserted into the vertebral body, it is removed, and a curved stylus is inserted to guide it to the center of the vertebral body. With proper placement, the stylus is retracted, and the cautery is placed and used to create a 1 cm spherical lesion (85˚C for 15 min). BVA involves targeting the basivertebral nerve within the vertebral body to disrupt pain signals (20). This procedure uses radiofrequency energy to ablate nerve fibers responsible for transmitting pain, providing long-lasting pain relief (20-23). BVA consistently reduces disability and pain levels across patient cohorts (21-25). The procedure has been reported to be effective for a significant proportion of patients, although some outliers do not experience any change in their disability levels (23,24). This technique is generally well-tolerated; however, potential side-effects include temporary discomfort at the treatment site, infection and nerve damage (23). These risks underscore the importance of precise techniques and patient selection (21,22).

## Patients and methods

### Patients and selection criteria

A retrospective analysis was conducted on patients who underwent either IDSI or BVA treatment for vertebrogenic CLBP separated into two groups. The technique employed in each group was performed exactly as aforementioned. The timeframe in which the present retrospective study was conducted was ~2 years, from January, 2022 to December, 2023. The patients in group 1 underwent IDSI treatment and this group consisted of 12 patients, while those in group 2 underwent BVA treatment and this group included 10 patients. Patient selection criteria included individuals aged from 18 to 70 years, males or females regardless of sex, who complained about persistent CLBP over the past ≥3 months at least, and having undergone lumbar spine MRI scan in which the findings (Modic changes type 1 and/or 2, vertebral endplate defects and endplate pathology) confirmed the vertebrogenic nature of the CLBP. In addition, in terms of inclusion criteria, the patients included had no notable comorbidities and major health issues, and had undergone only conservative treatment for their pain with *per os* medication and/or physiotherapy and no previous invasive treatment at any stage of the symptoms. On the contrary, the exclusion criteria included patients aged <18 years or >70 years of age, patients with no MRI findings that were associated with vertebrogenic CLBP and patients with symptoms lasting for <3 months. Furthermore, patients that were excluded from the study were individuals that had previously undergone any type of invasive and not conservative-only treatment for CLBP and patients with considerable comorbidities or major health issues. The patients were randomly allocated into the two groups, and no group-matching by age, sex, comorbidities or symptom duration was carried out. There were no criteria employed to determine whether a patient received the IDSI treatment or the BVA technique. There were no variations in the ablation protocol in the second group. ODI scores before and after treatment were collected to assess the impact of these interventions on disability and pain levels (16). The procedures were performed by the same physicians each time. Follow-up duration of the patients was 6 months in total, while patients were clinically evaluated at 1 week, 1 month, 3 months and 6 months following the intervention, respectively. The ODI score at 3 months post-intervention was utilized as post-treatment value in the statistical analysis as the improvement measurement time. No notable adverse events were observed in all the patients involved in the study during treatment and follow-up.

All procedures performed in studies involving human participants were in accordance with the ethical standards of the institutional and/or national research committee. The present study was approved by the Ethics Committee of KAT Attica General Hospital, Athens, Greece (Ref. no. 20869). Written informed consent was obtained from all patients included in the study.

### Statistical analysis

Comparisons of pre- and posttest outcomes for each treatment method were made using repeated measures ANOVA. The 2 patients who exhibited no improvement were excluded from the analysis (1 patient from each group). Furthermore, another reason for the exclusion of theses 2 patients was limited follow-up and incomplete data. All assumptions for the test were met (26). To improve the statistical power of the analysis when comparing the efficacy of each intervention against the other, the two methods were compared with ANCOVA, which provides the relative improvement of each treatment method compared with a normalized baseline. As per the prerequisite, all assumptions were met (27).

## Results

Both treatments revealed very promising results in reducing CLBP to levels practically considered complete resolution. In the present study sample, there were only two outliers, one for each method, which reported no difference in symptoms. These patients were excluded from the analysis due to the possible misdiagnosis of discogenic pain as the origin of their symptoms. At this point, it is imperative to underline that excluding 2 patients who were non-responsive may have led to bias in the outcomes and may overstate the effectiveness of the treatments. Notwithstanding, even in the event that those patients were finally included, both treatments resulted in a >90% reduction in pain and disability. In the analysis in the present study, as demonstrated by the data in Tables I and II, an improvement of <50% was considered subjectively moderate. A value >50% was considered notable, and a value <30% was considered as non-notable.

**Figure 1:**
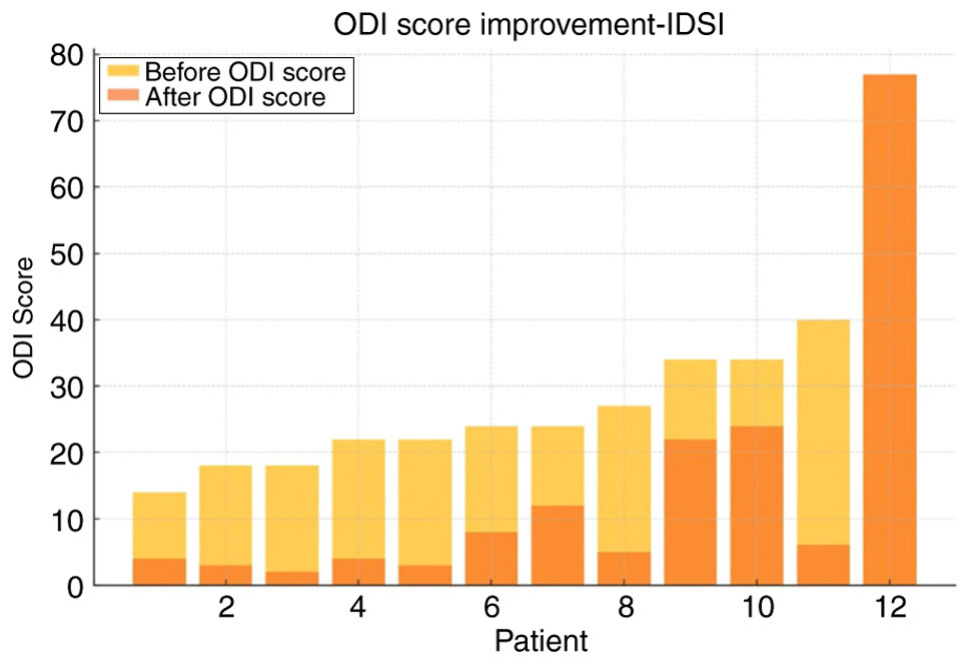
ODI score improvement for patients treated with IDSI. ODI, Oswestry Disability Index; IDSI, intradiscal steroid injection.

**Figure 2:**
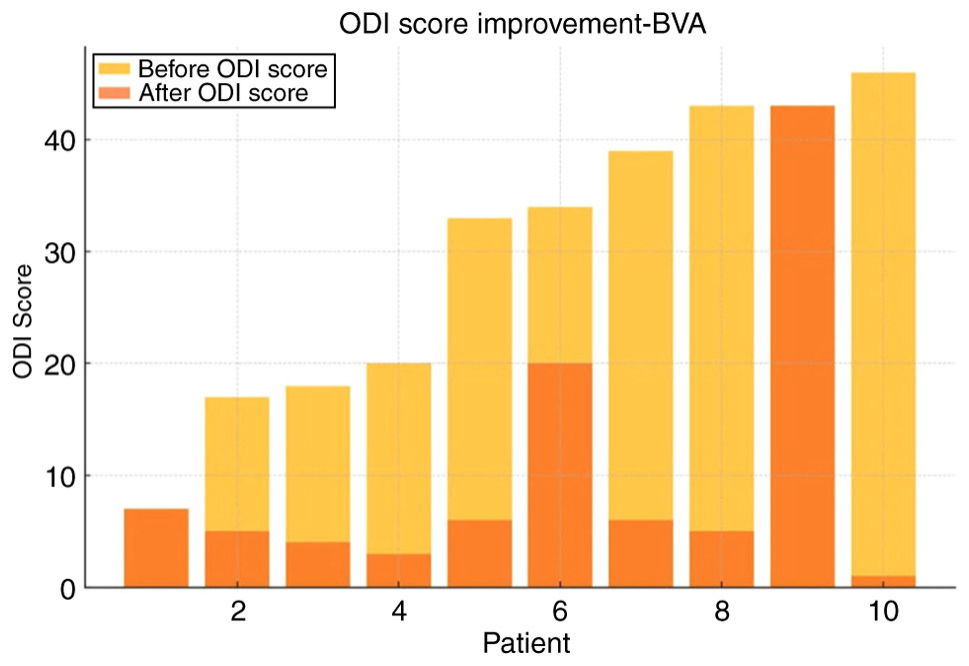
ODI score improvement for patients treated with BVA. ODI, Oswestry Disability Index; BVA, basivertebral nerve radiofrequency ablation.

**Figure 3:**
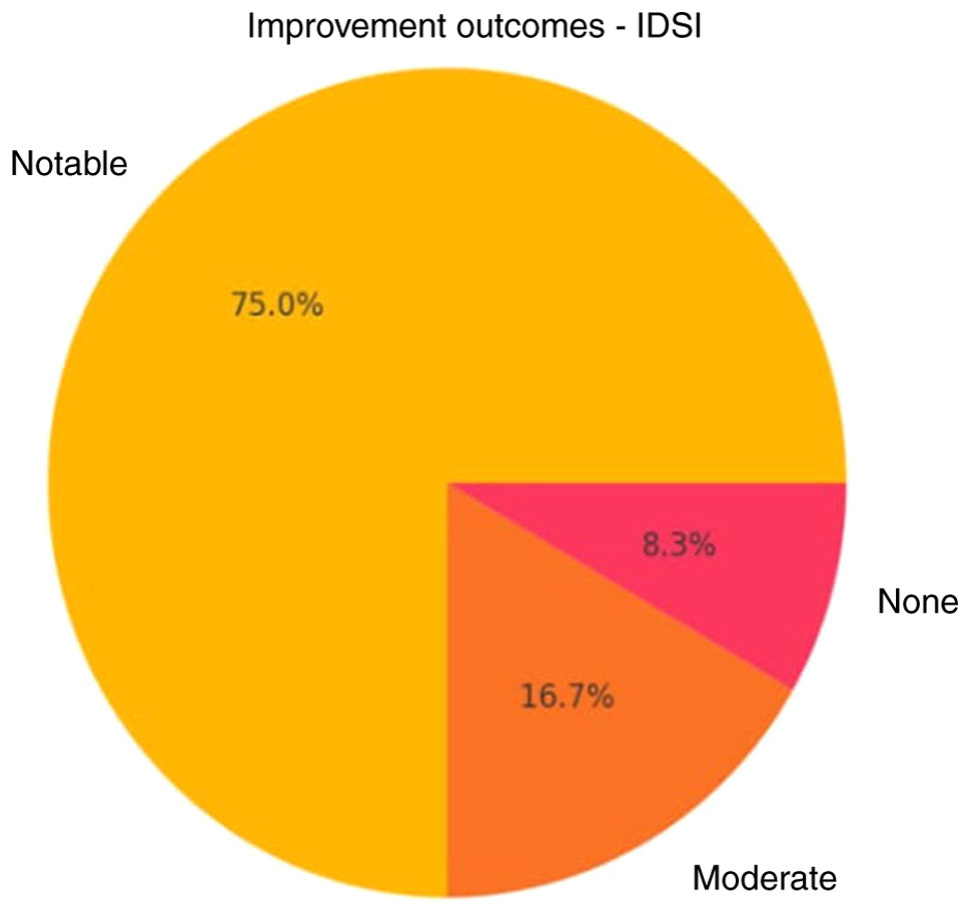
Improvement outcomes of patients treated with IDSI. IDSI, intradiscal steroid injection.

**Figure 4:**
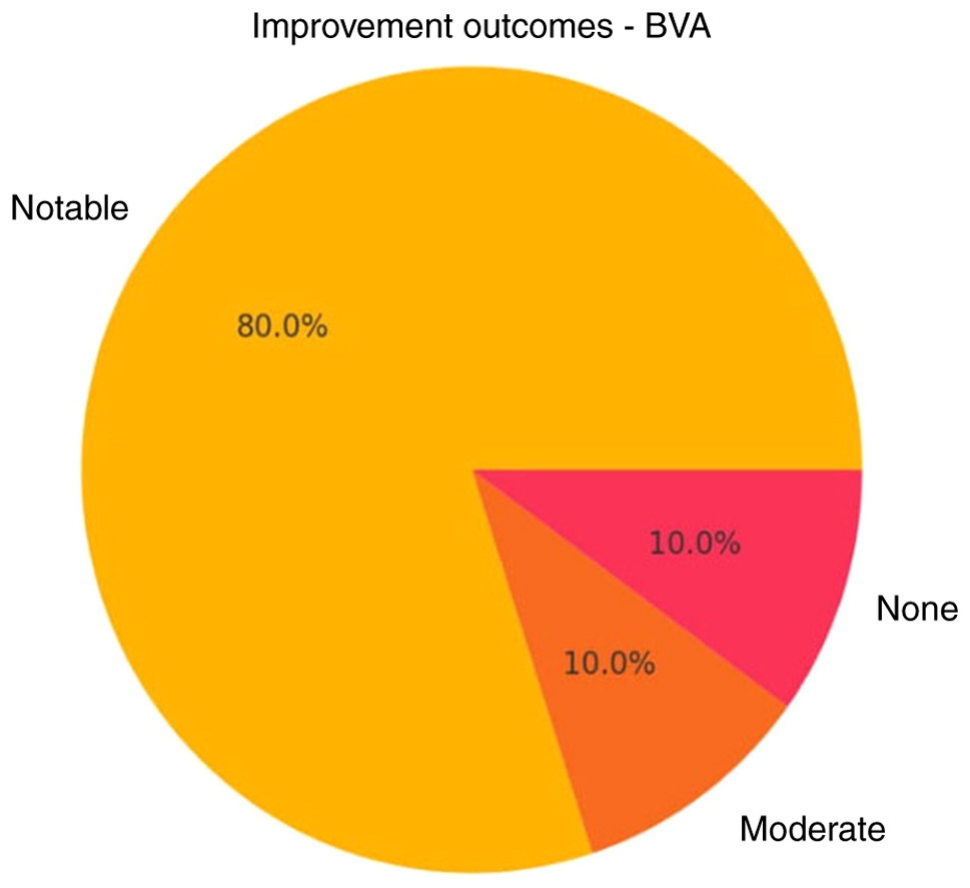
Improvement outcomes of patients treated with BVA. BVA, basivertebral nerve radiofrequency ablation.

For IDSI (Table I), the mean pre-treatment ODI was 29.9±16.5, which significantly decreased to 14.8±20.4 post-treatment (P=0.0001), representing an absolute reduction of 16.5 and a 66% relative improvement (Figs. 1 and 2). Of note, only 2 patients achieved moderate improvement (<50%). BVA treatment also demonstrated satisfactory results in reducing CLBP (Table II). In the BVA group (n=10), the mean ODI score significantly decreased from 30.1±13.6 before treatment to 10.1±12.6 after treatment (P=0.0023). When both groups were analyzed together, the overall baseline ODI mean was 28.5±15.2, which was reduced to 6.3±8.7 post-treatment, corresponding to a 78% mean reduction in disability. Notably, 90% of the patients experienced significant improvement (>50%) (Figs. 3 and 4).

Covariate analysis (ANCOVA) was conducted to control for potential confounding factors. The adjusted pre-treatment means were 26.7±14.8 for both groups. Covariates utilized in ANCOVA were baseline ODI scores, anxiety that was assessed using the Beck Anxiety Inventory (BAI) (mean ± SD: 12.4±3.5), fear-avoidance score using the Fear Avoidance Beliefs Questionnaire (FABQ) (18.9±5.2), age (47.2±8.1 years), sex (male:female, 9:13) and BMI (26.8±3.4 kg/m^2^). The adjusted post-treatment mean values were 8.7±6.1 for IDSI and 5.9±4.8 for BVA, with a statistically significant between-group difference (P=0.046). Overall, there appears to be a small improvement in BVA in the present study; however, owing to the small sample size of our patients, the statistical power of our research was 40%, which is not sufficient-enough for highly credible outcomes.

Both interventions in the present study resulted in a notable improvement for >80% of the patients treated and at least a moderate reduction in disability (between 30 and 50%) for >90% of them (Figs. 2 and 3). Furthermore, a minimal, yet considerable advantage was demonstrated in the analysis for BVA vs. IDSI, with the obvious caveat of the low statistical power of the research. Notably, both treatments resulted in post-treatment ODI scores of <10, 75% for IDSI and 80% for BVA, which translates to the almost complete resolution of symptoms (Figs. 1 and 2). Another noteworthy element is that 2 patients (1 patient from each group) exhibited no difference pre- and post-intervention, which may be attributed to unknown factors or that the treatments themselves could be ineffective in a percentage of patients (Fig. 1, Fig. 2, Fig. 3 and Fig. 4); it would be of interest to evaluate this finding in a larger patient sample.

Consequently, both IDSI and BVA could be considered viable and highly recommended options for immediate pain reduction in patients with CLBP; however, due to the methodological weaknesses of the present study, the findings can be considered as hypothesis-generating.

## Discussion

The comparative analysis of IDSI and BVA for the treatment of vertebrogenic CLBP can provide several critical insights. Both treatments have demonstrated efficacy in reducing pain and disability; however, their mechanisms and clinical outcomes vary, highlighting the need for a personalized approach in managing CLBP. The mechanisms of action for IDSI and BVA are distinct and crucial; although they are used for the same pathologic entity (CLBP), both demonstrate similar and high efficacy. IDPI utilizes the anti-inflammatory properties of locally infused corticosteroids in the body of the intervertebral disc (9,10).

Contrariwise, BVA targets the nerve pathways responsible for pain transmission on the vertebral endplates themselves, providing a more direct approach to pain relief. This method has exhibited consistent results across a broader range of patients, making it a reliable option for many patients (21-25). The direct disruption of pain signals can provide long-lasting relief, which is crucial for managing chronic conditions (4,5).

The difference in both the area and mechanism of treatment, compared with the similar effectiveness of IDSI and BVA, raises the question of which pain receptors are involved and what exactly the pathologoanatomical substrate of vertebrogenic CLBP is. If either disc or vertebral pain receptors are prevalent in CLBP, then the expected result of one of the treatments used herein would clearly be highly superior to that of the other, which was not the case reported in the present study or in the existing literature. As a result, further large-scale studies are warranted to investigate and differentiate the factors associated with CLBP.

Both treatments may trigger potential side-effects that need to be carefully managed. The side-effects of IDSIs, such as transient pain and increased blood sugar levels, require monitoring, particularly in patients with diabetes (9,10). BVAs, while generally well-tolerated, carry risks of nerve damage and infection, necessitating precise techniques and patient education about potential risks (23-25). Clinicians should weigh the benefits and risks of each treatment option, considering patient preferences and the likelihood of response. The selection between IDSI and BVA should be made by a comprehensive clinical assessment, ensuring that the selected treatment aligns with the specific condition and goals of the patient. The presence of outliers in both treatment groups suggests that neither IDSI nor BVA is universally effective for all patients. It should be emphasized that the exclusion of these patients represents a methodological flaw that can distort the reported outcomes. The variability in responses highlights the requirement for careful patient selection on the basis of specific clinical criteria. Factors, such as the nature of the pain, previous treatment responses and patient comorbidities should guide treatment decisions. Personalized treatment plans can optimize outcomes by matching patients with the most appropriate intervention. Given the various notable limitations of the present study, it is not facile to propose a standard treatment algorithm for vertebrogenic CLBP. Nonetheless, it can be denoted that these two interventions can be utilized in patients aged 18-70 years with no considerable comorbidities and persistent LBP for a period of >3 months where conservative treatment has failed, whist the selection of employing either IDSI or BVA as treatment of choice lies in the attending physician as they both seem as viable fruitful treatment options. In the case of the failure of one method, the attending physician may consider utilizing the other treatment method as an alternative; however, this practice can be the objective of further research.

Further research is essential to refine these treatments and identify predictors of response. Perusing the existing literature, no other study could be retrieved comparing these two treatment methods for CLBP, whilst only a limited number of review articles could be found presenting the various minimally invasive treatments for CLBP that already exist. Hence, prospective studies with larger sample sizes and randomized controlled trials are vital and could provide more definitive evidence on the comparative effectiveness of IDSI and BVA. Additionally, exploring combination therapies and adjunctive treatments may offer new avenues for patients who do not respond to either intervention alone.

For a more balanced interpretation of the outcomes, it is vitally important to acknowledge the copious major limitations of the present study which include the following: The retrospective design of the study, the small sample size, the exclusion of outliers, the absence of a placebo or sham control group, methodological flaws, reliance on subjective data (ODI scores), limited follow-up duration and low statistical power. At this point, the necessity for further larger-scale studies in this field is accentuated, as the present study can be considered as thought-provoking material.

In conclusion, both IDSI and intra-osseous BVA appeared to effectively reduce disability. The IDSIs demonstrated a higher percentage of complete symptom resolution, whereas BVAs provided more consistent significant improvements across patients. Despite potential side-effects, both treatments can provide substantial pain relief, potentially underscoring the significance of personalized treatment plans. Notwithstanding, in spite of both treatments exhibiting potential, taking into account the various considerable limitations of the present small-scale study, the findings should be regarded as hypothesis-generating, highlighting the requirement for further pertinent research in this area.

## Figures and Tables

**Table I:** Clinical outcomes of patients treated with intraarticular depo-methylprednisolone injection.

Patient no.	Sex (F/M)	Age (years)	BMI	Anxiety score (BAI)	Fear-avoidance score (FABQ)	Follow-up (months)	Initial disability level	ODI score before treatment	ODI score after treatment	Improvement outcome
1	M	51	22	15	22	18	Minimal	14	4	Notable
2	M	43	28	22	28	22	Minimal	18	3	Notable
3	F	61	30	25	29	6	Minimal	18	2	Notable
4	M	55	25	17	20	12	Moderate	22	4	Notable
5	F	40	21	20	28	20	Moderate	22	3	Notable
6	F	49	26	18	22	24	Moderate	24	8	Notable
7	M	58	20	18	25	10	Moderate	24	12	Notable
8	F	44	22	23	30	18	Moderate	27	5	Notable
9	F	59	31	26	32	12	Moderate	34	22	Moderate
10	M	46	27	14	26	6	Moderate	34	24	Moderate
11	M	43	25	15	23	14	Moderate	40	6	Notable
12	M	65	25	17	25	4	Crippling	77	77	None

M, male; F, female; BMI, body mass index; BAI, Beck Anxiety Inventory; FABQ, Fear Avoidance Beliefs Questionnaire; ODI, Oswestry Disability Index.

**Table II:** Clinical outcomes of patients treated with intra-osseous basivertebral nerve radiofrequency ablation.

Patient no.	Sex (F/M)	Age (years)	BMI	Anxiety score (BAI)	Fear-avoidance score (FABQ)	Follow-up (months)	Initial disability level	ODI score before treatment	ODI score after treatment	Improvement outcome
1	M	39	20	20	25	6	Minimal	7	7	Notable
2	M	48	26	25	31	18	Minimal	17	5	Notable
3	F	44	25	27	31	10	Minimal	18	4	Notable
4	M	62	25	22	24	22	Minimal	20	3	Notable
5	F	55	23	30	28	16	Moderate	33	6	Notable
6	F	58	30	26	25	4	Moderate	34	20	Moderate
7	M	44	20	16	22	24	Moderate	39	6	Notable
8	F	51	22	19	24	16	Severe	43	5	Notable
9	M	55	29	18	27	6	Severe	43	43	None
10	M	60	26	25	32	12	Severe	46	1	Notable

M, male; F, female; BMI, body mass index; BAI, Beck Anxiety Inventory; FABQ, Fear Avoidance Beliefs Questionnaire; ODI, Oswestry Disability Index.

## Data Availability

The data generated in the present study may be requested from the corresponding author.

## References

[1] Hoy D, Bain C, Williams G, March L, Brooks P, Blyth F, Woolf A, Vos T, Buchbinder R (2012). A systematic review of the global prevalence of low back pain. Arthritis Rheum.

[2] Shmagel A, Foley R, Ibrahim H (2016). Epidemiology of chronic low back pain in US adults: Data from the 2009-2010 National Health and Nutrition Examination Survey. Arthritis Care Res (Hoboken).

[3] Yang G, Liao W, Shen M, Mei H (2018). Insight into neural mechanisms underlying discogenic back pain. J Int Med Res.

[4] Ohtori S, Inoue G, Miyagi M, Takahashi K (2015). Pathomechanisms of discogenic low back pain in humans and animal models. Spine J.

[5] Conger A, Smuck M, Truumees E, Lotz JC, DePalma MJ, McCormick ZL (2022). Vertebrogenic pain: A paradigm shift in diagnosis and treatment of axial low back pain. Pain Med.

[6] Lorio MP, Beall DP, Calodney AK, Lewandrowski KU, Block JE, Mekhail N (2023). Defining the patient with lumbar discogenic pain: Real-world implications for diagnosis and effective clinical management. J Pers Med.

[7] Abel F, Altorfer FCS, Rohatgi V, Gibbs W, Chazen JL (2024). Imaging of Discogenic and vertebrogenic pain. Radiol Clin North Am.

[8] Tieppo Francio V, Sherwood D, Twohey E, Barndt B, Pagan-Rosado R, Eubanks J, Sayed D (2021). Developments in minimally invasive surgical options for vertebral pain: Basivertebral nerve Ablation-A narrative review. J Pain Res.

[9] Miller S, Caragea M, Carson D, McFarland MM, Teramoto M, Cushman DM, Cooper AN, Burnham T, McCormick ZL, Conger A (2024). The effectiveness of intradiscal corticosteroid injection for the treatment of chronic discovertebral low back pain: A systematic review. Pain Med.

[10] Hashemi M, Poorfarokh M, Mohajerani SA, Jalili P, Akhyani V, Barikani A, Farivar F (2014). Injection of intradiscal O_2_-O_3_ to reduce pain and disability of patients with low back pain due to prolapsed lumbar disk. Anesth Pain Med.

[11] Khalil JG, Smuck M, Koreckij T, Keel J, Beall D, Goodman B, Kalapos P, Nguyen D, Garfin S (2019). A prospective, randomized, multicenter study of intraosseous basivertebral nerve ablation for the treatment of chronic low back pain. Spine J.

[12] Michalik A, Conger A, Smuck M, Maus TP, McCormick ZL (2021). Intraosseous basivertebral nerve radiofrequency ablation for the treatment of vertebral body endplate low back pain: Current evidence and future directions. Pain Med.

[13] Fairbank JC, Pynsent PB (2000). The oswestry disability index. Spine (Phila Pa 1976).

[14] Johansson A, Hao J, Sjölund B (1990). Local corticosteroid application blocks transmission in normal nociceptive C-fibers. Acta Anesthesiol Scand.

[15] Ramesh G, Meisner OC, Philipp MT (2015). Anti-inflammatory effects of dexamethasone and meloxicam on *Borrelia burgdorferi*-induced inflammation in neuronal cultures of dorsal root ganglia and myelinating cells of the peripheral nervous system. J Neuroinflammation.

[16] Cao P, Jiang L, Zhuang C, Yang Y, Zhang Z, Chen W, Zheng T (2011). Intradiscal injection therapy for degenerative chronic discogenic low back pain with end plate Modic changes. Spine J.

[17] Nguyen C, Boutron I, Baron G, Sanchez K, Palazzo C, Benchimol R, Paris G, James-Belin É, Lefèvre-Colau MM, Beaudreuil J (2017). Intradiscal glucocorticoid injection for patients with chronic low back pain associated with active discopathy: A randomized trial. Ann Intern Med.

[18] Khot A, Bowditch M, Powell J, Sharp D (2004). The use of intradiscal steroid therapy for lumbar spinal discogenic pain: A randomized controlled trial. Spine (Phila Pa 1976).

[19] Tavares I, Thomas E, Cyteval C, Picot MC, Manna F, Macioce V, Laffont I, Thouvenin Y, Viala P, Larbi A (2021). Intradiscal glucocorticoids injection in chronic low back pain with active discopathy: A randomized controlled study. Ann Phys Rehabil Med.

[20] McCormick ZL, Sperry BP, Boody BS, Hirsch JA, Conger A, Harper K, Lotz JC, Burnham TR (2022). Pain location and exacerbating activities associated with treatment success following basivertebral nerve ablation: An aggregated cohort study of multicenter prospective clinical trial data. Pain Med.

[21] Becker S, Hadjipavlou A, Heggeness MH (2017). Ablation of the basivertebral nerve for treatment of back pain: A clinical study. Spine J.

[22] Fischgrund JS, Rhyne A, Franke J, Sasso R, Kitchel S, Bae H, Yeung C, Truumees E, Schaufele M, Yuan P (2018). Intraosseous basivertebral nerve ablation for the treatment of chronic low back pain: A prospective randomized double-blind sham-controlled multi-center study. Eur. Spine J.

[23] DSmuck M, Khalil J, Barrette K, Hirsch JA, Kreiner S, Koreckij T, Garfin S, Mekhail N (2021). Prospective, randomized, multicenter study of intraosseous basivertebral nerve ablation for the treatment of chronic low back pain: 12-month results. Reg Anesth Pain Med.

[24] Truumees E, Macadaeg K, Pena E, Arbuckle J, Gentile J, Funk R, Singh D, Vinayek S (2019). A prospective, open-label, single-arm, multicenter study of intraosseous basivertebral nerve ablation for the treatment of chronic low back pain. Eur Spine J.

[25] Macadaeg K, Truumees E, Boody B, Pena E, Arbuckle J, Gentile J, Funk R, Singh D, Vinayek S (2020). A prospective, single arm study of intraosseous basivertebral nerve ablation for the treatment of chronic low back pain: 12-month results. N Am Spine Soc J.

[26] Cohen J

[27] Borm GF, Fransen J, Lemmens WA (2007). A simple sample size formula for analysis of covariance in randomized clinical trials. J Clin Epidemiol.

